# Anodal Transcranial Pulsed Current Stimulation: The Effects of Pulse Duration on Corticospinal Excitability

**DOI:** 10.1371/journal.pone.0131779

**Published:** 2015-07-15

**Authors:** Shapour Jaberzadeh, Andisheh Bastani, Maryam Zoghi, Prue Morgan, Paul B. Fitzgerald

**Affiliations:** 1 Department of Physiotherapy, School of Primary Health Care, Faculty of Medicine, Nursing and Health Sciences, Monash University, Melbourne, Australia; 2 Department of Medicine, Royal Melbourne Hospital, The University of Melbourne, Melbourne, Australia; 3 Monash Alfred Psychiatry Research Centre, Monash University Central Clinical School and The Alfred, Melbourne, Australia; University of Montreal, CANADA

## Abstract

The aim is to investigate the effects of pulse duration (PD) on the modulatory effects of transcranial pulsed current (tPCS) on corticospinal excitability (CSE). CSE of the dominant primary motor cortex (M1) of right first dorsal interosseous muscle was assessed by motor evoked potentials, before, immediately, 10, 20 and 30 minutes after application of five experimental conditions: 1) anodal transcranial direct current stimulation (a-tDCS), 2) a-tPCS with 125 ms pulse duartion (a-tPCS_PD = 125_), 3) a-tPCS with 250 ms pulse duration (a-tPCS_PD = 250_), 4) a-tPCS with 500 ms pulse duration (a-tPCS_PD = 500_) and 5) sham a-tPCS. The total charges were kept constant in all experimental conditions except sham condition. Post-hoc comparisons indicated that a-tPCS_PD = 500_ produced larger CSE compared to a-tPCS_PD = 125_ (*P*<0.0001), a-tPCS_PD = 250_ (*P* = 0.009) and a-tDCS (*P* = 0.008). Also, there was no significant difference between a-tPCS_PD = 250_ and a-tDCS on CSE changes (*P*>0.05). All conditions except a-tPCS_PD = 125_ showed a significant difference to the sham group (*P*<0.006). All participants tolerated the applied currents. It could be concluded that a-tPCS with a PD of 500ms induces largest CSE changes, however further studies are required to identify optimal values.

## Introduction

Non-invasive induction of neuroplastic changes by transcranial stimulation techniques have been increasingly used in recent years. Transcranial direct current stimulation (tDCS) is a well-known neuromodulatory technique in which the direction of corticospinal excitability (CSE) changes depends on the polarity of the active electrode [1]. Literature indicates that the size of induced changes depends on current intensity used during tDCS application [1, 2].

In a recent study Jaberzadeh et al. (2014) demonstrated that conversion of direct current into unidirectional pulsatile current increases its efficacy for enhancement of CSE. The new paradigm was called transcranial pulsed current stimulation (tPCS)[3].

**Fig 1:**
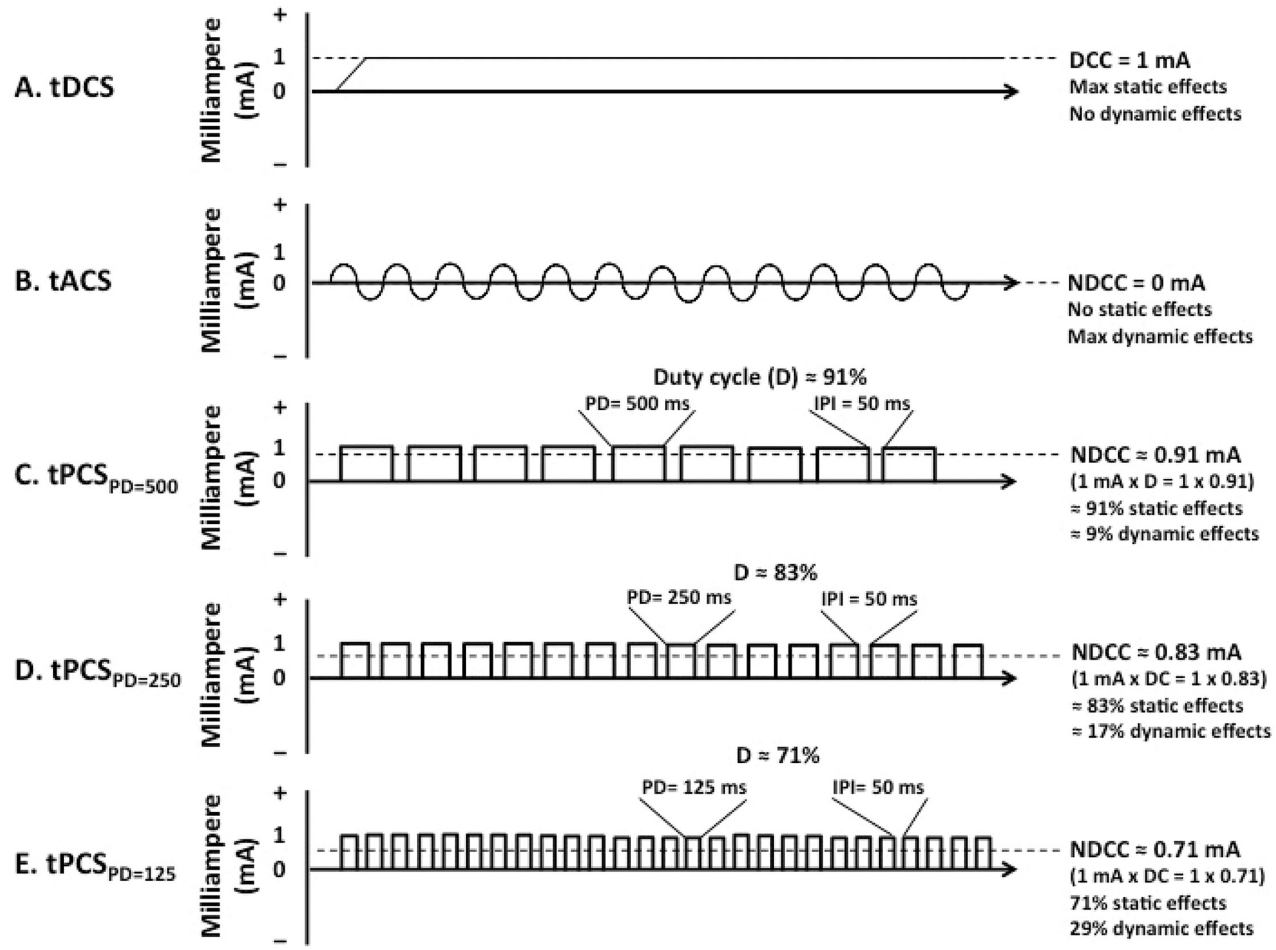
Direct, alternative and pulsatile currents. (a) tDCS; transcranial direct current stimulation, (b) tACS: transcranial alternating current stimulation, (c) tPCS _PD = 125_: transcranial pulsed current stimulation with 125 ms pulse duration, (d) tPCS _PD = 250_: transcranial pulsed current stimulation with 250 ms pulse duration, and (e) tPCS _PD = 500_: transcranial pulsed current stimulation with 500 ms pulse duration. DCC: direct current component; NDCC: net direct current component, PD: Pulse duration, IPI: Inter pulse interval, D: Duty cycle

In this paradigm, the direct current was interrupted to take advantage of two extra parameters, “pulse duration” (PD) and “inter-pulse interval (IPI)” (Fig 1). Compared to continuous flow of direct current in anodal tDCS (a-tDCS), anodal tPCS (a-tPCS) involves unidirectional flow of positive pulses, separated by a predetermined IPI. Dependent upon the duration and amplitude of pulses and IPI, tPCS produces different degrees of net direct current component (NDCC) [3]. Similar to tDCS, tPCS also involves the application of very low-amplitude currents (less than 2mA) via surface electrodes over the scalp and the current is well tolerated by healthy individuals [3]. However, unlike tDCS, due to the interrupted nature of tPCS, the presence of lights flashing in the eyes (retinal phosphene) is expected during application of this current [4].

Transcranial alternating current stimulation (tACS) is another neuromodulatory paradigm which has been introduced to modulate human cortical excitability [5–9]. This is a balanced current consisting of alternating biphasic pulses with equal electric charges. As a result, the average value of the voltage or current over application time is zero. Compared to tDCS, tACS allows manipulation of CSE not only based on intensity, but also based on the frequency of the applied current. Unlike tDCS where its excitatory or inhibitory effects are polarity dependent, tACS effects are determined by the frequency of the current [6, 9] and are not polarity dependent (Fig 1). In addition, sinusoidal tDCS (s-tDCS) [5] or slow oscillatory tDCS (so-tDCS) [10, 11] are modified versions of tACS where the alternative currents are added to a DC offset.

While the physiological mechanisms behind the effects of tPCS are not well understood, it was assumed that tPCS also induces its effects not only by polarity-dependent modulation of the baseline activity of the neurons in the primary motor cortex (M1), but also through the on–off effects of the pulses on M1 neurons. The introduction of tPCS raises a number of interesting and important questions about the role of these parameters in induction of CSE changes. For example it is unclear what is the optimal PD or IPI for induction of maximum CSE changes in M1 or which combination of PD or IPI induces maximum CSE changes.

Our previous research [3] indicated that with identical PDs, shorter IPIs induce larger changes in CSE. Unlike IPI, there is no published evidence to support the role of PD in induction of CSE changes. Therefore the primary objective of the current study was to investigate the effects of pulse duration on the modulatory effects of tPCS on M1 CSE. Based on the finding of our previous tPCS study [3], it was hypothesized that: 1. Both a-tDCS and a-tPCS of M1 would induce a CSE increase, which would remain significant up to 30 minutes post intervention and 2. Compared to conventional a-tDCS, which only affects the brain based on its static effects, a-tPCS, with both static and dynamic effects would induce larger changes in CSE irrespective of different PDs; 3. Sham a-tPCS of M1 will not change M1 CSE.

## Methods and Materials

### Subjects

Eleven healthy participants, (6 women, 5 men) with mean age 33.2 ± 7.21 (range 20–51 years) participated in this study. The sample size with a power of 80% was calculated based on a pilot study with 6 participants.[12] All participants were right-handed according to the 10 item version of the Edinburgh Handedness Inventory (88.3 ± 5.4) [13]. Prior to the experiments, all participants completed the Adult Safety Screening Questionnaire [14] to determine their suitability for TMS. Participants were informed of the experimental procedures and gave their written informed consent according to the declaration of Helsinki. All experimental procedures were approved by Monash University Human Ethics Committee. Each participant was tested at the same time of the day to counteract daytime variation.

### Study design and procedure

**Fig 2:**
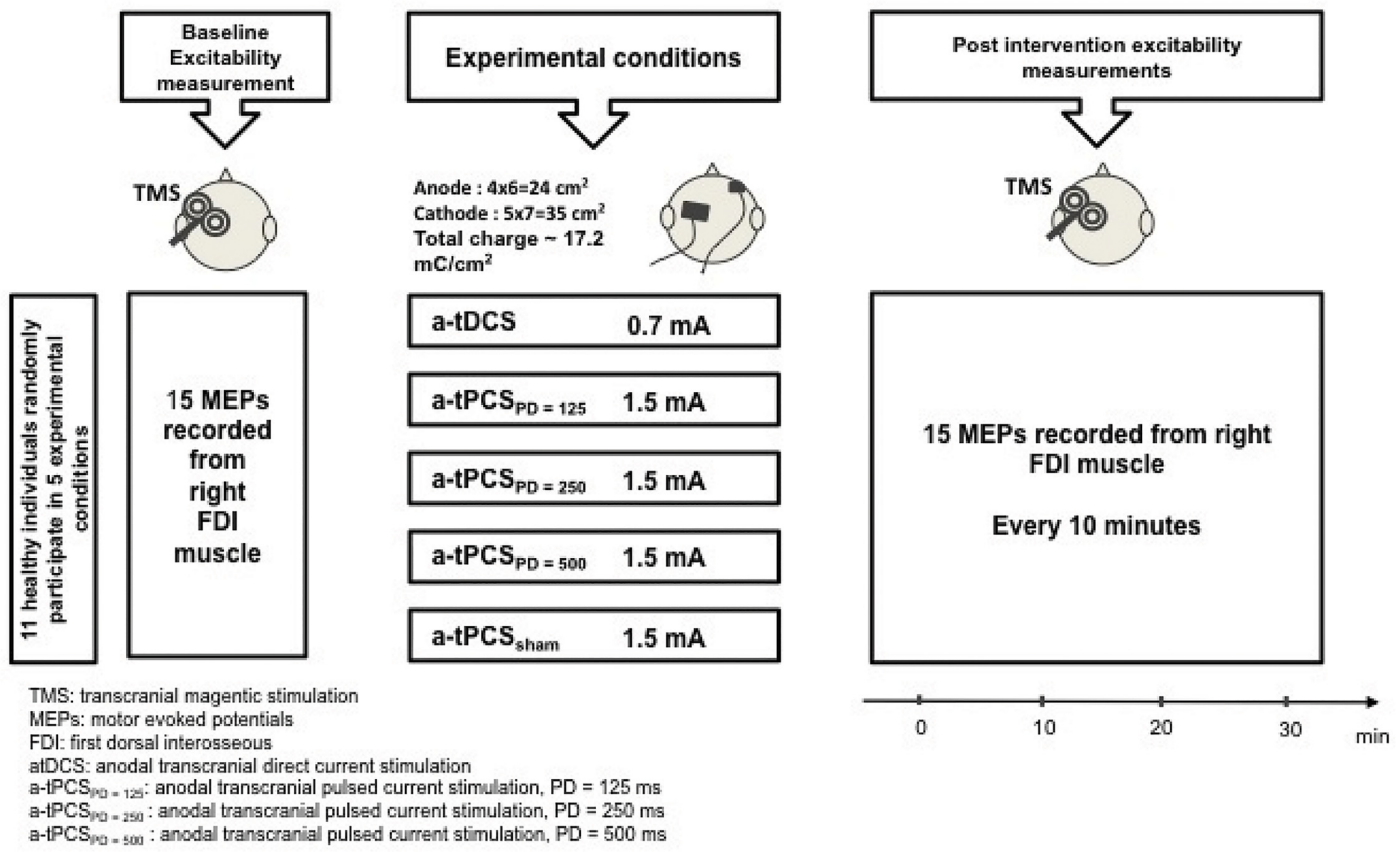
Study design for the comparison of different current types on CSE.

A single-blinded randomized sham-controlled cross-over design with 72 hours of washout period was used in this study. Participants were tested before, immediately after, and every 10 minutes up to 30 minutes after the end of 5 different experimental conditions (Fig 2): 1. a-tDCS, 2. a-tPCS _PD = 125ms,_ 3. a-tPCS _PD = 250ms,_ 4. a-tPCS _PD = 500ms_, and 5. Sham a-tPCS. Participants were blinded to the different stimulation conditions. The order in which the experimental conditions were conducted was randomized between participants.

### Application of a-tDCS

An Intelect Advanced Therapy System (Chattanooga, USA) was used to deliver a-tDCS through a pair of saline-soaked surface sponge electrodes. The active electrode (anode, 24 cm^2^) was placed over the left M1 for the right first dorsal interosseous (FDI) muscle as identified by TMS, and the cathode electrode (35 cm^2^) was placed over the right contralateral supraorbital area [1]. The electrodes were fixed with two straps. a-tDCS was applied continuously for 10 minutes with a current intensity of 0.7 mA (total charge ~ 17 mC/ cm^2^). The total charge was calculated based on the following formula:

$$TC=\frac{SI}{ES}\times TSD$$

Where TC is the total charge (mili-coulomb per centimeter squared, mC/cm^2^), SI is the stimulus intensity (miliampere, mA), ES is electrode size (cm^2^) and TSD is the total stimulation duration.

### Application of a-tPCS

The same device was used for delivery of a-tPCS through a pair of saline-soaked surface sponge electrodes. The anode (24 cm^2^) was placed over the left M1 for the right FDI muscle as identified by TMS, and the cathode (35 cm^2^) was placed over the right contralateral supraorbital area [1]. The impedance between the electrodes and the skin was kept below 10 kΩ. The waveform of the stimulation was unidirectional, pulsed and rectangular. The a-tPCS was delivered with the following parameters for a-tPCS_PD = 125_: current intensity: 1.5 mA, pulse duration: 125 ms, IPI: 50 ms and with a total duration of 20 minutes (total charge ~ 17 mC/ cm^2^). For a-tPCS_PD = 250_ it was: current intensity: 1.5 mA, pulse duration: 250 ms, IPI: 50 ms and with total duration of 10 minutes (total charge ~ 17 mC/ cm^2^) and for a-tPCS_PD = 500_ it was: current intensity: 1.5 mA, pulse duration: 500 ms, IPI: 50 ms and with total duration of 5 minutes (total charge ~ 17 mC/ cm^2^). The total charge for a-tPCS applications were calculated based on the following formula:

$$TC=\frac{SI}{ES}\times PD\times NP$$

Where TC is the total charge (mili-coulomb per centimeter squared, mC/cm^2^), SI is the stimulus intensity (miliampere, mA), ES is the electrode size (cm^2^), PD is the pulse duration (mSec) and NP is the number of pulses.

The above different parameters were chosen to keep the total charge constant between all conditions [10] rather than changing the stimulation intensities which would be less comfortable for participants. Therefore, based on the nature of the pulsed current, the inter-pulse intervals increased the duration of the study.

### Application of sham a-tPCS

For the sham stimulation, the electrodes were placed in the same positions as for experimental conditions 1–4; however, delivery of a-tPCS (a-tPCS_PD = 500_) was terminated after 30 seconds of stimulation. Hence participants felt initial sensations, but received no current for the remainder of the stimulation period (9 minutes and 30 seconds mins). This procedure permitted the blinding of participants for the respective stimulation condition [15].

### Measurement of side effects

All participants completed a questionnaire during and after completion of the experimental conditions. The questionnaire contained rating scales for the presence and severity of side effects such as tingling, itching, burning sensations under the electrodes [16] and other discomfort including eye flashing, headache and pain during and after all experimental conditions. All participants ranked the unpleasantness of any scalp sensations using numeric analogue scales (NAS) (e.g, 0 = no tingling to 10 = worst tingling sensation possible).

### Measurement of CSE by TMS

Participants were seated upright and comfortable with their head and neck supported by a headrest. Single-pulse magnetic stimuli were carried using a Magstim 200^2^ (Magstim Company Limited, UK) stimulator with a flat 70 mm figure-of-eight magnetic coil. Using the international 10–20 system, the vertex (C_z_) point was measured and marked to be used as a reference [2, 17]. The magnetic coil was placed over the left hemisphere (cortex), contralateral to the target muscles. The orientation of the coil was set at an angle of 45° to the midline and tangential to the scalp, such that the induced current flowed in a posterior-anterior direction. To find the optimal site of stimulation, the coil was moved around the M1 of FDI muscle to induce the largest motor evoked potential (MEP) responses. Then the coil position was marked with a marker on the scalp to be used for the remainder of the testing. The resting motor threshold (RMT) was defined as the minimal stimulus intensity that evoked 3 MEPs in a series of 6 with the amplitude of at least 50 μV [18–20] from the hotspot of the FDI muscle. The RMT for each participant was determined by increasing and decreasing stimulus intensity in 1–2% intervals until MEPs of appropriate size were elicited. For all further MEP measurement, the TMS intensity was set at 120% (1.2 times) of each individual’s RMT. Twenty MEP were elicited to assess CSE at each time point. The last fifteen MEPs were included in data analysis. The stimulus intensity remained constant throughout the experimental conditions for each participant.

### Electromyography (EMG) recording

Participants were seated in an adjustable podiatry chair with their forearm pronated and the wrist joint in a neutral position, resting on the armrest of the chair. To ensure good surface contact and reduce skin resistance, a standard skin preparation procedure of cleaning and abrading was performed for each site of electrode placement [17, 21, 22]. MEPs were recorded from the right FDI muscle at rest, using pre-gelled self-adhesive bipolar Ag/AgCl disposable surface electrodes with an inter-electrode distance of 3 cm, measured from the centre of the electrodes. The location of the FDI muscle was determined based on anatomical landmarks [23] and also observation of muscle contraction in the testing position [24]. The accuracy of EMG electrode placement was verified by asking the participant to maximally contract the FDI muscle while the investigator monitored online EMG activity. The ground electrode was placed ipsilaterally on the styloid process of the ulnar bone [25]. Electrodes were secured by tape. All raw EMG signals were band pass filtered (10–1000 Hz), amplified (×1000) and sampled at 2000 Hz, and were collected on PC running commercially-available software (Chart software, ADinstrument, Australia) via a laboratory analogue-digital interface (The PowerLab 8/30, ADinstrument, Australia). Peak-to-peak amplitude of MEPs was detected and measured automatically using a custom designed macro in Powerlab 8/30 software after each magnetic stimulus.

### Data management and statistical analysis

In this study, 15 MEPs were averaged off-line [26] for each time point before and after the intervention for all experimental conditions. All post intervention MEP amplitudes were normalised to the baseline value.

Baseline MEP amplitudes and RMT of the respective conditions were tested using one-way repeated measure ANOVA to see whether the baseline MEPs or RMT were identical in all conditions. This test was also carried-out on the mean values from NAS test to analyze the perceived sensation differences between different conditions.

A two-way repeated measure ANOVA was used to evaluate the effects of different current types on MEPs’ amplitude over time. The first within-subject independent factor was “current types” with five levels. The second independent factor was “time” with five levels. Mauchly's sphericity test was used to validate an assumption of repeated measures factor ANOVA. Greenhouse-Geisser corrected significance values were used when sphericity was lacking. Post-hoc comparisons were performed using the Bonferroni adjustment for multiple comparisons when appropriate. As an additional analysis, it was assessed whether sham and active stimulations were distinguishable. The participants were asked to indicate if they thought the stimulation was either active or sham at the end of their participation in all testing conditions. Data were analyzed using Pearson’s chi-square. The results were considered significant at the level of *P* < 0.05 for all statistical analyses. Bonferroni corrections were used as appropriate. All results are presented as the mean ± standard error of mean (SEM) and statistical analyses were performed using SPSS software version 20.

## Results

### Comparison of sensations in different conditions

All participants tolerated the applied currents in different experimental conditions with no interruption of experimental procedures due to adverse or side effects of the applied currents. Table 1 summarizes the numeric value means ± SEM for reported side-effects under the anode and cathode during a-tDCS, a-tPCS and sham a-tPCS. The mean values of side effects are all low indicating their mild nature. Lights flashing was experienced by 54% of the participants during a-tPCS_PD = 125_, a-tPCS_PD = 250_, a-tPCS_PD = 500_ and sham a-tPCS in a frequency dependent manner. There were no side effects reported by participants after the conclusion of experimental sessions. There were no reports of burning sensations, pain or headaches during or after a-tDCS or a-tPCS applications. The results of Bonferroni post-hoc comparisons for sensations are listed in Table 2.

**Table 1:** Numeric sensation scores (0 = no sensation to 10 = worst sensation imaginable) reported by participants during experimental conditions. Scores are reported as mean ± SEM.

	Sensation	Anode	Cathode
**a-tDCS**	Itching	3.39 ± 0.29	2.39 ± 0.19
	Tingling	2.54 ± 0.18	1.84 ± 0.45
	Eye flashing	None	
**a-tPCS**_**PD = 125**_	Itching	1.09 ± 0.09	0.42 ± 0.10
	Tingling	0.98 ± 0.12	0.33 ± 0.07
	Eye flashing	2.93 ± 0.58	
**a-tPCS**_**PD = = 250**_	Itching	1.06 ± 0.19	0.24 ± 0.07
	Tingling	0.39 ± 0.15	0.18 ± 0.06
	Eye flashing	1.37 ± 0.47	
**a-tPCS**_**PD = 500**_	Itching	1.39 ± 0.21	0.30 ± 0.07
	Tingling	0.68 ± 0.31	0.28 ± 0.09
	Eye flashing	2.40 ± 0.47	
**Sham a-tPCS**	Itching	0.81 ± 0.27	0
	Tingling	0.51 ± 0.12	0
	Eye flashing	0.57 ± 0.15	

**Table 2:** Comparison of side effects between a-tDCS, a-tPCS_PD = 125_, a-tPCS_PD = 250_, a-tPCS_PD = 500_ and sham conditions. The results were considered significant at the level of *P* < 0.05.

	Comparison between conditions	Anode		Cathode	
		F value	*P* value	F-value	*P* value
Sensation		(4,50)		(4,50)	
**Itching**	tDCS-tPCS_PD = 500_	21.50	*P* < 0.001	79.75	*P* < 0.001
	tDCS-tPCS_PD = 250_		*P* < 0.001		*P* < 0.001
	tDCS- tPCS_PD = 125_		*P* < 0.001		*P* < 0.001
	tDCS-Sham		*P* < 0.001		*P* < 0.001
	tPCS_PD = 125—_tPCS_PD = 500_		*P* = 1.00		*P* = 1.00
	tPCS_PD = 125—_tPCS_PD = 250_		*P* = 1.00		*P* = 1.00
	tPCS_PD = 500—_tPCS_PD = 250_		*P* = 1.00		*P* = 1.00
	tPCS_PD = 125_-Sham		*P* = 1.00		*P* = 0.08
	tPCS_PD = 500_ –Sham		*P* = 0.78		*P* = 0.55
	tPCS_PD = 250_ –Sham		*P* = 1.00		*P* = 1.00
**Tingling**	tDCS-tPCS_PD = 500_	20.19	*P* < 0.001	71.94	*P* < 0.001
	tDCS-tPCS_PD = 250_		*P* < 0.001		*P* < 0.001
	tDCS- tPCS_PD = 125_		*P* < 0.001		*P* < 0.001
	tDCS-Sham		*P* < 0.001		*P* < 0.001
	tPCS_PD = 125—_tPCS_PD = 500_		*P* = 1.00		*P* = 1.00
	tPCS_PD = 125—_tPCS_PD = 250_		*P* = 1.00		*P* = 1.00
	tPCS_PD = 500—_tPCS_PD = 250_		*P* = 1.00		*P* = 1.00
	tPCS_PD = 125_-Sham		*P* = 1.00		*P* = 0.10
	tPCS_PD = 500_ –Sham		*P* = 0.78		*P* = 0.25
	tPCS_PD = 250_ –Sham		*P* = 1.00		*P* = 1.00
**Eye flashing**	tDCS-tPCS_PD = 500_	9.16	*P* = 0.001	-	-
	tDCS-tPCS_PD = 250_		*P* = 0.19		-
	tDCS- tPCS_PD = 125_		*P* < 0.001		-
	tDCS-Sham		*P* = 1.00		-
	tPCS_PD = 125—_tPCS_PD = 500_		*P* = 1.00		-
	tPCS_PD = 125—_tPCS_PD = 250_		*P* = 0.08		-
	tPCS_PD = 500—_tPCS_PD = 250_		*P* = 0.77		-
	tPCS_PD = 125_-Sham		*P* = 0.001		-
	tPCS_PD = 500_ –Sham		*P* = 0.024		-
	tPCS_PD = 250_ –Sham		*P* = 1.00		-

### Participants’ awareness of active versus sham conditions

Table 3 provides the data on participants’ awareness of the stimulation being used in testing experimental conditions. Pearson’s chi square was not significant (χ^2^ (8df) = 5.66, *P* = 0.68), suggesting that participants could not accurately verify the type of stimulation received and they were successfully blinded. Overall, the percentage of participants who correctly guessed the active condition was 27.2% (excluding ‘cannot say’ responders) and 81.8% (including ‘cannot say’ responders).

**Table 3:** Number of subject’s who guessed the active or sham stimulation conditions.

		Actual testing conditions (n = 11)					
		a-tDCS	a-tPCS_PD = 125_	a-tPCS_PD = 250_	a-tPCS_PD = 500_	Sham	Totals
**Perceived stimulation**	Active	2	5	3	3	2	15
	Sham	3	2	3	1	1	10
	Cannot say	6	4	5	7	8	30
	Totals	11	11	11	11	11	55

### Comparison of different conditions

One-way repeated measure ANOVA showed that baseline MEPs’ amplitude (F_(4,40)_ = 1.68, *P* = 0.32, partial ŋ^2^ = 0.18) and RMT (F(1,10) = 1.41, *P* = 0.36, partial ŋ^2^ = 0.19) were identical for all conditions.

The assumption of sphericity had been met for time (W = 0.154, df = 9, *P* = 0.078), current type (W = 0.356, df = 9, *P* = 0.474) and current type × time interaction (W = 0.000, df = 135, *P* > 0.05).

**Fig 3:**
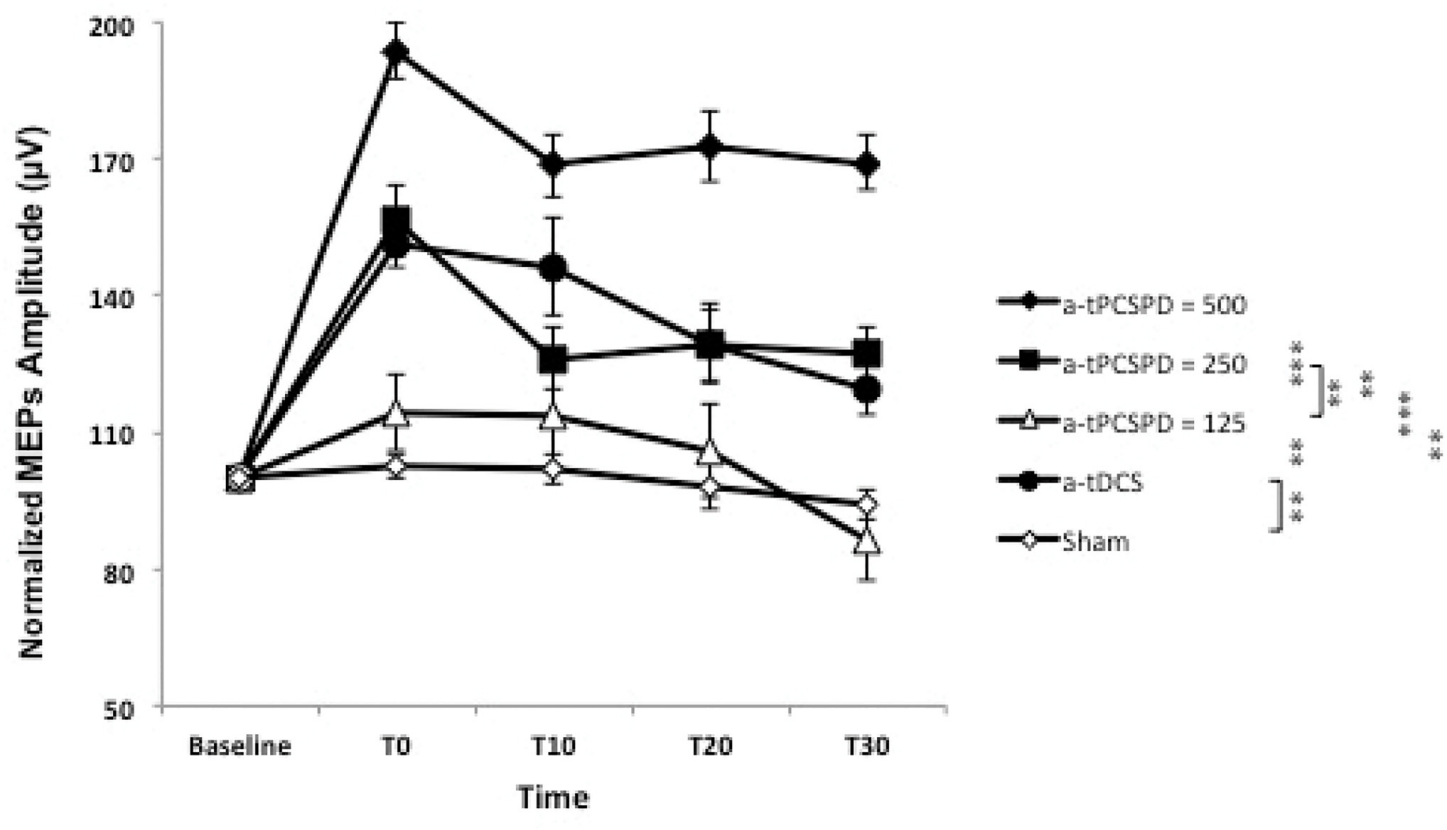
The effects of different current types on the lasting effects and slope of decrease for MEPs’ amplitude over time. Filled symbols indicate significant deviation of the post transcranial stimulation MEP amplitudes relative to the baseline; the asterisks mark significant differences between different testing conditions. Data are reported as mean ± SEM

The results of the two-way repeated measures ANOVA showed significant main effects of current type (F(4,40) = 36.009, *P* < 0.001, partial ŋ^2^ = 0.78), time (F(4, 40) = 38.02, *P* < 0.001, partial ŋ^2^ = 0.79) and the interaction of current type × time (F(16, 160) = 6.99, *P* < 0.001, partial ŋ^2^ = 0.41) (Table 4) (Fig 3).

**Table 4:** Significant interactions between mean MEPs of different conditions in different time points. The results were considered significant at the level of *P* ≤ 0.001.

		Time points			
		T0	T10	T20	T30
**Stimulation Conditions Comparison**	tDCS-tPCS_PD = 500_	0.001*	0.148	0.003	0.000058**
	tDCS-tPCS_PD = 250_	0.525	0.020	0.99	0.151
	tDCS- tPCS_PD = 125_	0.001*	0.110	0.017	0.010
	tDCS-Sham	0.000069**	0.001*	0.003	0.00029**
	tPCS_PD = 125—_tPCS_PD = 500_	0.000035**	0.003	0.001*	0.000004**
	tPCS_PD = 125—_tPCS_PD = 250_	0.004	0.378	0.091	0.004
	tPCS_PD = 500—_tPCS_PD = 250_	0.11	0.005	0.013	0.002
	tPCS_PD = 125_-Sham	0.426	0.232	0.273	0.544
	tPCS_PD = 500_-Sham	2.09E-09**	0.00003**	0.000036**	0.000002**
	tPCS_PD = 250_-Sham	0.000134**	0.012	0.015	0.000009**

Post-hoc comparisons for the main effect of the “Current type” indicated that a-tPCS_PD = 500_ produced larger increases in CSE compared to a-tPCS_PD = 125_ (Mean = 56.71, SEM = 6.29; *P* < 0.0001), a-tPCS_PD = 250_ (Mean = 32.37, SEM = 6.96; *P* = 0.009) and a-tDCS (Mean = 31.44, SEM = 6.63; *P* = 0.008). However, there was no significant difference between a-tPCS_PD = 250_ and a-tDCS on CSE changes (*P* > 0.05) (Fig 3). a-tPCS_PD = 250_ and a-tDCS also produced larger CSE compared to a-tPCS_PD = 125_ (Mean = 24.34, SEM = 6.11; *P* = 0.02) and sham a-tDCS (Mean = 25.26, SEM = 6.90; *P* = 0.04) respectively. The results also showed that there was no significant differences between a-tPCS_PD = 125_ and sham tPCS on CSE changes (*P* > 0.05). However, all other three current types (a-tDCS, a-tPCS_PD = 250_ and a-tPCS_PD = 500_) showed significant changes of CSE compared to the sham stimulation (*P* < 0.001).

The lasting effects of changes in different conditions are presented in Fig 3, where a-tDCS, a-tPCS_PD = 250_, and a-tPCS_PD = 500_ resulted in significant excitability enhancement lasting at least for 30 minutes (*P* < 0.005). However, following both sham a-tPCS and a-tPCS_PD = 125_, there were no significant changes in the MEPs amplitude in different time points (*P* > 0.05).

A paired sample t-test with Bonferroni correction was used to compare mean MEPs in different time points between different conditions (Table 4). The statistical significance between repeated post stimulation readings of MEP amplitudes within each condition is shown in Table 5.

**Table 5:** Significant differences between repeated post stimulation readings of MEP amplitudes within each condition. The results were considered significant at the level of *P* ≤ 0.001.

		Time points					
		T0-T10	T0-T20	T0-T30	T10-T20	T10-T30	T20-T30
**Stimulation Conditions**	a-tDCS	0.47	0.006	0.001*	0.044	0.023	0.087
	a-tPCS_PD = 500_	0.009	0.035	0.018	0.259	0.905	0.497
	a-tPCS_PD = 250_	0.089	0.107	0.014	0.868	0.974	0.885
	a-tPCS_PD = 125_	0.984	0.519	0.028	0.515	0.086	0.171
	Sham	0.132	0.039	0.002	0.349	0.412	0.992

## Discussion

### General considerations

In general, the current study supports the use of a-tPCS, with minimal or no side effects, in healthy participants. In line with the findings of Jaberzadeh et al. (2014), the participants tolerated a-tPCS better than conventional a-tDCS. No adverse effects were recorded or resulted in termination of the experiments. The reported side effects in the current study are consistent with ones reported earlier and include tingling and itchiness sensations [27]. These scalp sensations could occur due to the electrochemical effects of NDCC under the electrodes [28–30]. These side effects were minimized during and after the application of a-tPCSs regardless of PD parameter. In general, the tDCS-induced sensations were perceived more frequently and more strongly than a-tPCS or sham a-tPCS conditions for all of the sensations reported. Also, participants were unable to distinguish whether the stimulation was real or sham.

### The phosphene

In line with the findings of Jaberzadeh et al. (2014), application of a-tPCS, induced phosphene (light flashing) in 54% of participants’ eyes. The rate of these light flashings was correlated to the frequency of the applied pulses [3, 31]. This could be caused by high sensitivity of the retina to frequent on/off nature of the pulses during application of a-tPCS electrical stimulation [32]. The retinal phosphene was only present during application of a-tPCS and faded when the stimulation was completed.

### Comparison of different conditions

Firstly, it was hypothesized that both a-tDCS and a-tPCS of M1 would induce an increased CSE, which would remain significant up to 30 minutes post intervention. This hypothesis was partially supported by the findings in the present study. This increase was only found following a-tDCS and a-tPCS with longer PDs (_PD = 500_ and _PD = 250_). On the contrary, a-tPCS with shorter PD (_PD = 125_) failed to show any post intervention increase in CSE. Current knowledge on tPCS effects on the CSE changes is limited, and the mechanisms underpinning this effect are not yet understood. Different mechanisms may explain the results presented in this study. It is already established that the effect of a-tDCS is due to unchanged flow of direct current [20]. Conversely, unlike a-tDCS which changes CSE by tonic depolarization of the resting membrane potential, a-tPCS changes CSE by a combination of *tonic* and *phasic* effects of the applied pulses (See Jaberzadeh et al. 2014 for more details).

In the present study, to keep the IPI (50ms), intensity (1.5 mA) and total charge (~ 17 mC/ cm^2^) constant, the application time of a-tPCS and therefore the number of pulses per application were different. The application times of a- tPCS were 20, 10 and 5 minutes for PDs of 125 and 250 and 500 milliseconds, respectively. Interestingly, it was found that shorter application of a-tPCS, as in a-tPCS_PD500_, induced larger CSE changes than longer a-tPCS applications. The main question is how the prolonged stimulation period in PD-125 and PD-250 might contribute to the absence/weaker effect in comparison to other a-tPCS conditions? In exploring this question it should be noted that longer applications of a-tPCS, as in PD-125 and PD-250, apply lower NDCC (Fig 1). This indicates that stimulation with lower PDs produces lower tonic effects. This may show that the phasic, on-off, characteristics of the applied pulses is not the primary factor for induction of changes. Therefore, in line with our previous a-tPCS study [3], the current study also indicates that a combination of tonic and phasic effects of applied currents have an important role in the induction of CSE changes.

Secondly, it was hypothesised that, compared to conventional a-tDCS, a-tPCS with different PDs induces larger CSE changes. This hypothesis is also partially supported by the findings in the present study. The larger changes are only induced following a-tPCS with the longest PD (_PD = 500_). While a-tPCS _PD = 125_ failed to induce any CSE changes, the a-tPCS _PD = 250_ only induced CSE changes comparable to a-tDCS (Table 4).

In the current study, tPCS_PD = 500_ had the highest NDCC compared to a-tPCS _PD = 250_ and _PD = 125_ (Fig 1). This finding not only shows the significance of both tonic and phasic effects of the applied currents, but also indicates that there should be optimal combination of values for these tonic and phasic components of the currents. Based on the findings in the present study, we can conclude that a PD of 500ms with IPI of 50ms induces the largest CSE changes, and a PD of 125ms with IPI of 50ms induces no CSE changes. However it is not possible to draw any final conclusions regarding optimal values of PD and IPI for induction of the largest changes. Further studies are required to identify these optimal values.

Although the characteristics of the current used in this study are different to that of Bergmann et al. (2009) [10] and Groppa et al. (2010) [11], the results can be discussed in relation to excitability effects of so-tDCS which to some extent shares similar characteristics of dynamic and static effects with tPCS.

The rationale behind os-tDCS or tACS protocols is to interact with endogenous oscillatory cortical activity. In alignment with the findings of the current study, Bergmann [10] and Groppa’s [11] groups concluded that anodal so-tDCS and a-tDCS can induce comparable effects on CSE when the total current charge is matched. However, the results of the current study are in contrast to their findings that found no significant difference in the amount of CSE increase between a-tDCS and anodal so-tDCS. This discrepant finding could be explained by different stimulation durations (2 _ 20 min and 10 min vs. 5 min) and lower frequency (0.75 and 0.8 Hz vs. 1.8 Hz) that have been used by Bergmann et al. (2009) [10] and Groppa et al. (2010) [11], respectively.

The third hypothesis in the current study indicated that a-tPCS of M1 does not have any sham effects. The results of the present study support this hypothesis. This finding is in line with the finding of our previous a-tPCS study [3], which indicated the lack of placebo effects for this novel neuromodulatory technique.

### Study limitations

The findings in the current study must be considered in the context of the following limitations. Firstly, even though it has been shown that the induction of retinal phosphene on its own does not have any effect on CSE [3], its interaction with stimulation might have modulatory effects on CSE. Due to methodological limitations, this interaction was not evaluated in this study. Secondly, to keep the electrode size and total charge identical, the stimulus intensity and the length of tPCS application were variable in different experimental conditions. Therefore the findings in the current study should be interpreted with consideration that higher amplitudes will produce larger electrical field strength in deeper brain tissues, potentially resulting in effects on different neuronal populations. Thirdly, the small sample size limits generalizability of the findings. A fourth consideration is that the data were attained from a healthy population with no neurological disorders, limiting the potential to generalize findings to patients with neurological disorders. Finally, the effects were assessed on young participants. Older individuals may respond differently to a-tDCS or a-tPCS.

### Suggestions for future studies

This study evaluated the effects of three different PDs on the size of induced CSE in M1. Further investigation is required to identify the optimal PD of tPCS for generation of M1 CSE changes. The current study evaluated the lasting effects of a-tPCS over a time duration of up to 30 min. Further studies are required to extend the lasting effects of a-tPCS when applied for longer duration. The effects of different characteristics of pulsatile currents such as amplitude and frequency of the pulses, are additional areas which should be scientifically explored to define the optimal parameters for prolongation of tPCS lasting effects. In addition to cortical changes, future studies should also assess behavioural changes such as motor performance in both healthy individuals and patients with neurological conditions. Furthermore, to underline the mechanisms behind efficacy of a-tPCS, a mechanistic study of motor cortex excitability should be undertaken, by measuring silent period, intracortical inhibition, and facilitation, to assess the function of inhibitory and facilitatory neurotransmitters. Additional experiments including simultaneous assessment of EEG are required to refine tPCS as a method of frequency dependent brain stimulation.
